# Exploring the role of virtual reality in preparing emergency responders for mass casualty incidents

**DOI:** 10.1186/s13584-025-00681-9

**Published:** 2025-04-09

**Authors:** Alena Lochmannová

**Affiliations:** Department of Emergency Medicine, Diagnostic Disciplines and Public Health, Faculty of Health Care Studies, University of West Bohemia, Husova 11, 301 00 Pilsen, Czech Republic

**Keywords:** Virtual reality training, Mass casualty incidents, Paramedic training, Emergency preparedness, Crisis simulation

## Abstract

**Background:**

The increasing complexity of mass casualty incidents (MCIs) necessitates highly effective training for emergency responders. Traditional training methods, while effective in teaching core skills, often fail to replicate the dynamic, high-pressure environments responders face in real-world crises. Virtual reality (VR) offers a novel approach to emergency training, providing an immersive, controlled setting that can simulate real-life scenarios. This study explores the effectiveness of VR in training paramedic students for MCIs and compares the outcomes to those from conventional training methods.

**Methods:**

A comparative study was conducted with 37 paramedic students who underwent either VR-based training or conventional training using mannequins and real-world equipment. The VR application simulated a mass casualty car accident, focusing on triage and patient management. Both groups were assessed based on their performance in key areas, including the accuracy of situational reporting (METHANE), patient triage, heart rate monitoring, and perceived demand using the NASA Task Load Index (NASA-TLX).

**Results:**

The VR group demonstrated significantly lower mental demand (*p* < 0.001) and frustration levels (*p* = 0.021) compared to traditional training. However, task completion times were slower in the VR setting (*p* < 0.001), likely due to the interface's unfamiliarity. Accuracy in situational reporting was higher in VR (*p* = 0.002), while heart rate monitoring did not reveal a significant difference between the groups (*p* = 0.516). Although VR did not reduce temporal demand (*p* = 0.057), it showed potential for improving focus and precision in training. Error rates in triage were similar across both training methods (*p* = 0.882), indicating comparable performance levels in patient classification.

**Conclusions:**

VR presents a promising tool for training emergency responders, particularly in situations that require rapid upskilling, such as crises or wars. The ability to simulate realistic, high-pressure scenarios in a controlled environment can enhance both cognitive and emotional preparedness. Further research is necessary to optimize VR systems and interfaces, making them more efficient for real-time decision-making. As VR technology advances, it holds potential as a key component in future emergency preparedness strategies.

## Introduction

Unpredictable disasters and mass casualty incidents (MCIs), whether caused by human actions or natural forces, affect regions across the globe. In recent times, there has been a significant move away from relying on reactive measures after disasters occur, shifting towards proactive disaster preparedness. This approach is essential for reducing the overall impact on affected communities and for ensuring the greatest chance of saving lives [1]. In response to the growing need for effective disaster preparedness, high-fidelity virtual reality (VR) has emerged as a promising tool for simulating realistic and repeatable training scenarios. While VR training shows great potential, further evaluation is necessary to fully optimize its use. However, existing studies often focus on the general benefits of VR for skill acquisition but either lack direct comparisons between VR and traditional methods or provide only theoretical analyses without incorporating control groups [2]. This study specifically addresses these gaps by evaluating how VR training impacts cognitive load, decision-making accuracy, and overall preparedness in managing MCIs. By directly comparing VR and conventional approach, the research highlights VR's unique contributions and limitations in this specialized context.

Emergency professionals require training that ensures they can work safely and efficiently in high-pressure disaster situations [3]. VR has been recognized as an effective learning method that enhances individual performance, particularly by allowing users to practice specific skills and problem-solving techniques in a controlled, immersive environment [4]. This approach not only sharpens technical abilities but also builds the confidence needed to respond effectively in real-world emergencies [5].

One of the main benefits of VR training is that it offers a controlled and safe environment where participants can engage in realistic simulations without being exposed to the intense physical stressors often present in real-world scenarios [6]. This controlled setting allows learners to focus on skill development and problem-solving while maintaining a level of psychological and physical safety. To enhance realism, controlled stressors, such as background noises, visual distractions, or large crowds of bystanders, can be incorporated into the simulation [5, 7]. These elements help trainees experience situational pressure similar to real-world emergencies while ensuring the stress levels remain manageable and conducive to learning. By carefully calibrating these stressors, VR training can replicate high-pressure environments without overwhelming participants, ultimately improving both cognitive and emotional preparedness [8]. Monitoring physiological indicators, such as heart rate and perceived stress, provides additional insights into how trainees adapt to these conditions, ensuring that VR captures the complexities of high-stakes emergency situations [6].

The controlled nature of VR training has proven particularly advantageous in healthcare, offering tailored, immersive simulations that closely resemble real-life emergency scenarios. This approach allows healthcare professionals to practice and refine their skills in a safe, repeatable environment, enhancing their preparedness for critical situations such as MCIs [6]. Research has demonstrated that controlled, immersive training settings can improve overall performance and reduce mental strain, making VR an invaluable tool for emergency preparedness [3, 9].

VR training offers a unique opportunity to integrate conventional training methods with advanced virtual simulations. While VR systems can autonomously evaluate performance by generating score reports, the traditional debriefing process remains an essential component of the learning experience. This blend allows for a thorough review of participant actions, where automated insights from VR are complemented by expert feedback [10]. In this way, virtual reality serves as a bridge between technology-driven assessments and human-driven instruction, ensuring that learners not only receive immediate, data-driven evaluations but also gain deeper insights through personalized, expert-led debriefings [9].

VR has proven to be especially beneficial for preparing paramedics for fast response scenarios. It ensures that even reserve personnel can be rapidly upskilled to meet the demands of urgent deployments. This capability is vital during large-scale emergencies, where the need for well-trained personnel is immediate. The flexibility and adaptability of VR allow for quick, efficient training, making it an indispensable tool in large-scale crisis management [2].

In VR training, certain limitations can lead to a higher rate of errors, particularly in complex tasks like patient triage. One contributing factor is the lack of real-world sensory feedback, such as tactile interaction and environmental cues, which are crucial for making quick and accurate decisions in real-life scenarios [6]. Additionally, challenges with navigating the virtual environment in VR training can contribute to errors, particularly in patient triage [11]. Difficulties with maneuvering controls or adjusting to the virtual interface may lead to slower response times and incorrect decisions. Motion sickness, although rare, can also arise in some users, further complicating their ability to focus and fully engage with the simulation [12].

Trainees in virtual environments often find themselves needing to adjust to the unique challenges of the setting, which differs from real-world scenarios. This shift requires them to adopt new strategies for problem-solving and decision-making that may not align with what they would naturally use in a physical space [13]. The absence of tactile feedback, the reliance on virtual cues, and the use of simulation-specific tools all demand a different approach to tasks such as patient triage or emergency response [8]. Participants must reinterpret the environment and rely on alternative methods to assess situations and execute procedures. These strategic adjustments enhance cognitive flexibility [14] but can also add a layer of complexity, as they navigate both the limitations and benefits of the virtual medium.

Preparation for MCIs is essential for healthcare professionals, even though these events may occur less frequently than routine emergencies. MCIs present inherent complexity due to their highly variable nature. The challenges of managing such events arise from numerous factors, including the type of incident, the number of casualties, the availability of resources, as well as external conditions like weather and timing [15]. These factors make it extremely difficult to recreate realistic training environments for healthcare personnel, as the variability in MCIs is nearly impossible to predict or standardize in traditional training [16]. Designing an effective curriculum that prepares emergency responders to manage these diverse and unpredictable conditions is no small task. This is where VR proves to be an invaluable tool. Although recreating every possible scenario is challenging, VR can simulate a wide range of MCI conditions and variables, offering a more dynamic and adaptable training platform. By allowing healthcare workers to engage with realistic, though simulated, scenarios, VR provides the opportunity to practice and refine essential skills that are difficult to teach through conventional means [3]. This makes VR an essential supplement to traditional MCI training, ensuring that emergency personnel are better equipped to handle the unpredictable nature of real-world crises [17].

The primary objective of this study is to examine the role of VR in preparing emergency responders for MCIs. The research aims to determine how VR simulations can enhance the training process by providing realistic, high-pressure scenarios that mirror the complexities of real-world incidents. Specifically, the study evaluates whether VR training can improve the cognitive and decision-making abilities of paramedics, focusing on key factors such as error rates, response times, and overall efficiency in managing MCIs. While existing studies often highlight the potential of VR, this research builds on that foundation by providing empirical evidence through a controlled experimental design. By comparing VR with traditional training methods, the study offers practical insights into how VR can complement existing approaches, ensuring emergency personnel are better equipped for unpredictable, high-stakes situations.

## Method

This study is a mixed-method research project that examines the effectiveness of VR training compared to traditional training methods for preparing paramedic students to respond to MCIs. A comparative experimental design was employed with two independent groups: the experimental group, which underwent VR-based training, and the control group, which received traditional, in-person training with mannequins and real-world equipment.

The primary aim of the research is to evaluate how VR simulations can enhance practical skills and decision-making in high-pressure, emergency scenarios. By comparing performance metrics between an experimental group using VR and a control group using conventional training tools, the study seeks to determine whether immersive technologies can offer a significant advantage in medical education and crisis management.

### Participants

The study involved a total of 37 third-year university students in paramedicine, who had already completed 2 years of practical training in emergency medicine and were in their final year before entering full professional practice. The total number of participants represented the majority of third-year paramedic students enrolled in the program, with the sample size naturally limited by the size of the cohort. The intended sample size was approximately 50 participants, divided equally into two groups. Based on this plan, the power to detect a large effect size (Cohen's d = 0.8) using a t-test would have been the commonly targeted 80%. However, due to the voluntary nature of participation, only 15 and 22 participants remained in the two groups at the end of the study. For these group sizes, the power is lower, approximately 65%. This implies that the study is primarily powered to detect large or very large effects, if present. The division into the experimental and control groups was performed using a random number generator to ensure unbiased allocation. The smaller experimental group size reflected the increased time and technical demands associated with the VR training, whereas the larger control group ensured sufficient representation for statistical analysis. The study included 24 women and 13 men, reflecting the typical gender distribution among paramedic students. Statistical analysis confirmed no significant influence of gender on the study outcomes. Third-year students were specifically selected due to their prior experience with training in MCIs. Participation in the study was entirely voluntary, and participants were allowed to withdraw from the study at any point.

Before the study began, both groups completed a baseline questionnaire to assess their previous experience with emergency incidents and their subjective level of preparedness, as well as their familiarity with virtual reality.

### Experimental group

The virtual reality application used for the study was designed to simulate MCI, specifically focusing on the triage and management of patients at an accident scene. The application allowed users to independently navigate the training modules without needing external assistance. The VR system was created to provide a realistic training environment in which users could interact with elements typically found in an MCI scenario. It was developed using Unity3D and run on Valve Index VR headsets, ensuring high-quality interaction and immersion for the participants. The application also incorporated a pre-designed module structure, allowing step-by-step progress through scenarios, and featured automated feedback systems to guide participants in real-time.

The initial environment of the VR application was set in an ambulance garage, which served as a transition zone. Here, users could adjust their VR headsets, familiarize themselves with the environment, and prepare for the training modules without feeling rushed. They interacted with a virtual control panel to navigate through training modules and tasks. This preparatory phase ensured participants were comfortable with the VR interface before transitioning to the main scenario. Once ready, participants could proceed with the training scenarios.

The simulated scenario involved a car accident at night, designed to introduce additional complexity through reduced visibility and evening conditions. The decision to stage the scenario during evening hours was made to replicate the real-life challenges paramedics often face, where poor lighting conditions make decision-making more difficult. The virtual environment recreated a suburban area with the accident involving two vehicles. The simulation featured six to nine patients, and for the purposes of the experiment, both the control and experimental groups were exposed to six casualties. Each casualty was programmed with specific behavioral patterns, such as moving limbs, simulating unconsciousness, or verbally responding to inquiries. The application was programmed to include both adults and children. To ensure equal conditions for both the experimental and control groups, only adult casualties were used in this study, and the same type of injuries were maintained across both groups. This consistency allowed for accurate comparison between the two training methods while minimizing variability in injury complexity.

Participants interacted with various static and dynamic elements within the environment. Static elements included vehicles and medical equipment like oxygen tanks and bandages, which were strategically placed to reflect a realistic accident scene. The positioning of these elements was randomized across iterations to evaluate participants' adaptability and decision-making under varying environmental layouts. Dynamic elements involved six virtual patients with diverse but standardized injuries to ensure comparability between the groups. Each casualty displayed a specific injury pattern, supported by visual cues like bleeding, bruises, and immobilization needs, as well as auditory cues such as groaning or silence (indicative of unconsciousness). Smartwatches provided vital parameters like pulse, breathing rate, and capillary refill time, visible only briefly to replicate real-world constraints. Participants had to recall or note the data for patient evaluation. The temporary visibility of vital signs emphasized the importance of accurate and timely decision-making under pressure. Additionally, virtual patients' vital signs were programmed to deteriorate over time if critical interventions were delayed, requiring participants to prioritize tasks dynamically. Some of the patients could be interacted with through an option to ask questions, allowing participants to inquire about specifics of the patients' subjectively perceived health conditions.

The VR application included a training phase where participants were guided by a virtual instructor, represented by an avatar dressed as a paramedic colleague. The avatar provided real-time guidance and feedback to help participants navigate the scenario and practice key tasks. This phase ensured participants were familiar with the MCI protocol and the VR interface. In the subsequent testing phase, participants completed tasks independently, without the avatar's assistance. Performance was monitored in real-time, with data collected on the time taken to complete each task, the accuracy of triage decisions, and any errors made during the scenario.

Similarly, in the control group, participants first reviewed the key elements of responding to a mass casualty incident with the help of an instructor. Only after this review did they proceed to the practical simulation, ensuring that both groups received a structured and systematic approach to their training and testing.

The core training modules focused on two key components. First, participants practiced the METHANE protocol, where they were required to report the accident to a virtual dispatcher. This task was completed through a simulated tablet interface, allowing users to input the correct information step by step. In addition, participants used the START triage system to assess patients. They utilized virtual tools, including "smart watches," to measure vital signs such as pulse, breathing rate, and capillary refill time. Based on these measurements, participants categorized patients into one of four color-coded triage levels. Those patients who needed immediate treatment using the tools and equipment of the rescue backpack had to be treated immediately by the participants, otherwise their condition could worsen and they could even die, which would subsequently be shown in the final evaluation of the application.

### Control group

The control group underwent the same scenario-based training but using traditional methods in a physical classroom setting. Like the experimental group, their training began in a simulated ambulance environment, where participants prepared for the scenario. The lighting in the classroom was adjusted to simulate nighttime conditions with reduced visibility, ensuring comparable environmental factors for both groups. Instead of interacting with a virtual reality simulation, participants worked with mannequins that were represented by live actors, whose injuries were carefully crafted to mirror those used in the VR simulation. The injuries were typologically identical, ensuring that both groups encountered the same types of trauma and patient conditions.

An experienced paramedic instructor acted as a guide throughout the training, filling the same role as the virtual avatar in the VR environment. This instructor provided real-time feedback and gave detailed instructions on how to handle the multi-casualty traffic accident scenario, including patient triage using the START system and reporting the situation using the METHANE protocol. The participants practiced real-life first aid interventions, such as stopping major bleeding and managing airways, using actual medical tools.

In addition to guiding the training, the paramedic instructor also evaluated the participants’ performance. This evaluation was based on their actions during the simulation, how well they followed protocols, and how effectively they applied the necessary first aid interventions. Unlike in the VR environment, where these aspects were monitored automatically, the control group’s performance was manually assessed by the instructor.

The total duration of the training intervention was approximately 15–20 min for both groups, ensuring comparable training exposure.

### Data collection

Both the experimental and control groups were assessed using several key metrics. The primary measures of performance included the time to complete the METHANE report, the accuracy of triage classifications using the START system, the number of critical errors made during the scenario, and subjective workload evaluations using the NASA Task Load Index (NASA-TLX).

For the METHANE report, participants were timed from the moment they began reporting to the virtual or real dispatcher until the report was completed. The experimental group, using the VR system, had their time automatically recorded by the application, while in the control group, the instructor manually timed each participant.

In terms of triage accuracy, participants used the START system to classify patients into one of four color-coded triage levels based on their conditions.

Participants' critical errors were recorded, focusing on major mistakes made during the triage or treatment processes that could have negatively impacted patient outcomes, such as misclassifying patients or failing to apply life-saving interventions correctly.

Additionally, heart rates were monitored in the experimental group throughout the training using Garmin Venu Sq smartwatches to assess stress levels during the scenario. Finally, both groups completed the NASA Task Load Index (NASA-TLX) questionnaire post-training to evaluate their subjective workload, including mental, physical and temporal demands, performance, effort, and overall frustration.

In the experimental group, all performance data (e.g., task completion times, errors) was automatically logged by the VR system. In contrast, the control group's performance was manually evaluated by the paramedic instructor using predefined assessment criteria.

## Results

Statistical evaluation was conducted using RStudio (version 2022.12.0+353, R version 4.2.3), applying a 5% significance level. To test the assumption of normality before conducting t-tests and ANOVA, the Shapiro–Wilk test was used. If normality was not rejected, a t-test was applied to compare two independent groups. However, if normality was rejected in at least one group, the non-parametric Mann–Whitney U test was used instead. When comparing three or more independent groups, ANOVA was used if the normality assumption held and the ratio of the largest to smallest sample standard deviation did not exceed 3. If normality was rejected or the standard deviation ratio exceeded this threshold, the Kruskal–Wallis test was applied.

The statistical hypotheses for these tests compared either means or medians, depending on the test used. For post-hoc analysis, significant differences between specific groups were identified when the null hypothesis was rejected. Additionally, Fisher’s exact test was used to assess dependencies in contingency tables. In this case, the hypotheses were based on the presence or absence of an association between variables.

The study involved a total of 37 participants, divided into two groups: the control group (22 participants) and the experimental group (15 participants). The average age in both groups was 22, with a slight variation in the maximum ages, 26 in the control group and 27 in the experimental group. The gender distribution included 15 women in the control group compared to 9 women in the experimental group.

The main hypotheses tested during the study were aimed at evaluating various aspects of performance and stress experienced by participants in both VR and real-world training settings. The hypotheses were as follows:

### H1

Participants in the VR environment have lower heart rates compared to real-world training.

### H2

The temporal demand (as measured by NASA TLX) is lower in VR than in the real-world environment.

### H3

The mental demand (as measured by NASA TLX) is lower in the virtual environment compared to real-world training.

### H4

The level of frustration is lower in the VR environment compared to real-world training.

### H5

There is a higher error rate in patient triage in the virtual environment compared to the real-world environment.

### H6

The selection order of patients for treatment differs in the virtual environment compared to the real-world environment.

### Maximum heart rate and experience factors

Heart rate monitoring was conducted using Garmin Venu Sq smartwatches, but only during the test phase of the study. This phase allowed for an assessment of participants' physiological stress levels in response to the simulation of MCIs. The hypothesis (H1) suggested that participants in the VR environment would exhibit lower heart rates compared to those undergoing traditional training, indicating a less stressful environment.

The maximum heart rate was measured for both the control and experimental groups. Since normality was not met for the control group (*p* = 0.033) according to the Shapiro–Wilk test, a Mann–Whitney U test was employed to compare the groups. The results showed no significant difference between the groups (*p* = 0.516, Cliff’s delta = 0.13, 95% CI for difference [− 13, 9], the effect size is negligible), which led to the conclusion that both groups had similar maximum heart rates, allowing the subsequent analysis to combine the data from both groups.

When analysing the data by gender, no significant differences in maximum heart rate were found (*p* = 0.307, Cliff's delta = − 0.21, 95% CI for difference [− 15, 6], the effect size is negligible). Additionally, no significant differences were observed based on whether the probands had prior experience with death (*p* = 0.056, Cohen's d = 0.52, 95% CI for difference [− 0.23, 16.70], the effect size is moderate) or varying lengths of practice (*p* = 0.070, Cohen's d = − 0.60, 95% CI for difference [− 19.49, 0.82], the effect size is moderate), confirming the initial hypothesis that these factors did not significantly impact heart rate under stress.

Participants were asked to self-assess their fear of responding to MCI on a scale from 1 to 10. This measure aimed to capture their subjective perception of fear related to real-life MCI scenarios, rather than fear associated with the simulation or VR environment itself. The study examined how the fear of responding to an MCI influenced heart rate. Here, a Kruskal–Wallis test was used since normality was rejected in the group with moderate fear (*p* < 0.001). The results revealed a significant difference between groups (*p* = 0.042, eta2 = 0.127, the effect size is moderate), with higher fear levels correlating paradoxically with lower maximum heart rates. Specifically, those with moderate fear had median heart rate 112 (95% CI [110, 121]), while those with higher fear levels had median heart rate 101 (95% CI [95, 107]), challenging the expectation that higher stress would lead to higher heart rates.

Hypothesis H1 posited that the experimental group would have a lower heart rate due to the immersive nature of VR training. However, the results did not support this hypothesis. The experimental group’s heart rate did not significantly differ from the control group’s, indicating that VR did not reduce physiological stress responses compared to traditional training methods.

### Assessment of demand using NASA TLX

The NASA Task Load Index (NASA TLX) was used to assess six aspects of demand: mental demand, physical demand, temporal demand, performance, effort, and frustration. The results showed statistically significant differences between the experimental and control groups in two dimensions.

Participants in the VR group reported significantly lower mental demand compared to the control group (median 50 in the experimental group vs. 75 in the control group, *p* < 0.001, Cliff's d = − 0.67, 95% CI for difference [− 35, − 15], effect size is large). This reduction highlights the structured and guided nature of the VR environment, which may help participants focus on key tasks without the distractions common in real-world settings. Similarly, frustration levels were notably lower in the VR group (mean 34.7 vs. 52.0, *p* = 0.021, Cohen's d = − 0.79, 95% CI for difference [− 31.91, − 2.85], the effect size is moderate), suggesting that the immersive and controlled nature of VR contributed to a more positive training experience.

Although temporal demand was lower in the VR group (mean 41.7 vs. 55.9), the difference did not reach statistical significance (*p* = 0.057, Cohen's d = − 0.67, 95% CI for difference [− 28.91, 0.42], the effect size is moderate).Therefore, the hypothesis that temporal demand would be lower in the VR environment was not confirmed. This trend could reflect the potential of VR to enhance focus, but further refinement of the VR interface may be required to address issues such as task execution speed.

**Fig. 1:**
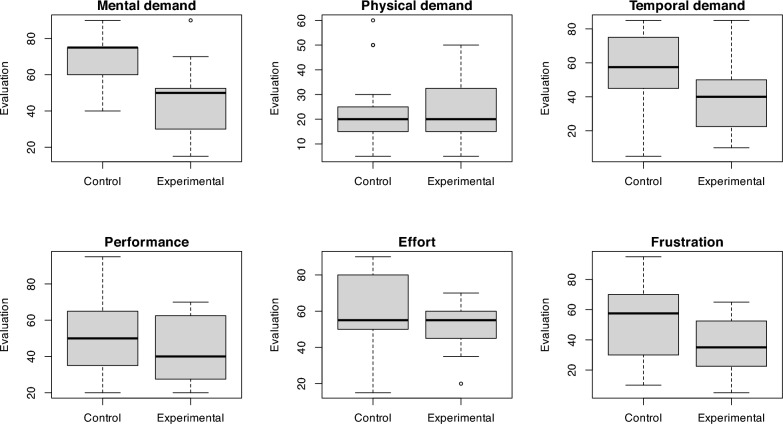
Characteristics of NASA TLX—Boxplots by Groups

Interestingly, no significant differences were observed in physical demand, performance, or effort, which may indicate that the core tasks in both environments required similar levels of physical and cognitive exertion. The different types of demand are shown for the experimental and control groups using boxplots in Fig. 1.

Hypothesis H2 assumed that participants in the virtual environment would experience lower temporal demand compared to those undergoing traditional training. Temporal demand was defined as the subjective feeling of pressure during task completion, not the actual time spent in training. It was expected that the reduced complexity and improved focus in VR would lead to a lower perceived pressure on participants. The results, however, showed that although participants in the experimental group reported lower temporal demand (mean 41.7) compared to the control group (mean 55.9), this difference did not reach statistical significance (*p* = 0.057), the effect size is moderate. Therefore, Hypothesis H2 was not confirmed. Despite the trend toward lower temporal demand in the VR environment, this result was not statistically strong enough to support the hypothesis.

Hypothesis H3 assumed that mental demand would be lower in the virtual environment than in traditional training. This assumption was based on the idea that VR provides a more interactive and guided environment, helping participants focus better on key tasks without the distractions of the real world, thus reducing cognitive load. The results supported this hypothesis, as mental demand was significantly lower in the VR environment (mean 44.3) compared to traditional training (mean 68.6) with high statistical significance (*p* < 0.001), the effect size is large. This confirms that VR does indeed reduce mental demand, indicating that participants faced fewer cognitive challenges during VR training.

Hypothesis H4 assumed that the level of frustration would be lower among participants who underwent training in the VR environment compared to those who underwent traditional training. This assumption was based on the idea that VR training is more immersive, intuitive, and less stressful, which should result in a lower level of frustration. The results confirmed this hypothesis, as participants in the experimental group reported significantly lower frustration levels (mean 34.7) compared to the control group (mean 52.0), with a p-value of 0.021, the effect size is moderate. This suggests that the virtual environment can help reduce frustration during training, leading to a more positive experience for participants.

### Analysis of METHANE reporting

The total time required to complete the METHANE report was significantly different between the two groups. In the experimental group, the average completion time was 281 s, compared to 207 s in the control group, a difference confirmed as statistically significant (*p* < 0.001, Cohen's d = 1.49, 95% CI for difference [36.91, 110.34], the effect size is large). While participants in the VR group took longer to complete the report, they achieved significantly higher accuracy, with an average score of 5.7 out of 6 compared to 4.7 in the control group (*p* = 0.002, Cramér's V = 0.62, the effect size is strong). This highlights a trade-off between speed and precision, with the immersive VR environment contributing to better retention of critical information and fewer reporting errors.

In real-world emergency scenarios, where the accuracy of situational reporting is paramount, the advantages of enhanced precision may outweigh the drawback of slower task execution. This indicates that VR training fosters meticulous reporting skills, which are essential for effective crisis management and decision-making in high-pressure situations.

### Error rates in patient triage

The analysis of patient triage error rates showed no significant difference between the experimental and control groups in terms of the number of misclassified patients. In the experimental group, which used VR, an average of 0.87 patients per participant were misclassified (ranging from 0 to 4 patients), while in the control group, which worked with traditional methods, an average of 0.64 patients per participant were misclassified (ranging from 0 to 2 patients). The results suggest that both groups exhibited similar error rates, even though the VR environment offered a completely different type of interaction with patients compared to the physical training setting with real models. In both environments, participants were required to make decisions based on key parameters such as heart rate and respiratory activity. Errors were primarily recorded in more complex cases where patients exhibited atypical symptoms, requiring quick decision-making.

Fisher’s exact test did not reveal any statistically significant difference in the error rates between the two groups (*p* = 0.882, Cramér's V = 0.20, the effect size is weak). This indicates that both the VR and traditional training environments provided participants with a comparable level of accuracy in patient triage. Based on these results, no significant difference in error rates was demonstrated between the groups, suggesting that the type of environment (virtual or real) did not significantly affect the overall number of errors during patient triage. Therefore, Hypothesis H5, which anticipated higher error rates in the virtual environment, was not supported.

### Approach to patient triage

In the experimental group, the perception of virtual reality was assessed based on subjective feedback from the participants. The study revealed that most participants felt that the VR environment provided a highly immersive experience. However, a small portion of participants reported discomfort, with symptoms such as nausea or blurred vision. Fisher’s exact test (*p* = 0.315, Cramér's V = 0.33, the effect size is moderate) confirmed that these issues were independent of gender. Additionally, participants within the experimental group generally indicated that the virtual environment felt real, but no statistically significant difference between men and women was found in terms of this perception.

Other tests also provided valuable insights. For instance, when probands were asked to evaluate their perception of errors during the scenarios, the experimental group showed more uncertainty about their performance, likely due to the constraints imposed by the VR environment, such as limitations on interacting directly with patients. This led participants to adopt a more systematic approach, which might have contributed to a higher perceived error rate, even though their objective performance was like that of the control group.

In the process of triage, the selection of the order in which patients are treated is a critical aspect, as it determines the effectiveness of the emergency response. The study aimed to investigate whether the choice of patient prioritization in a virtual environment differs from that in real-life scenarios. Both experimental and control group participants were asked to explain what factors influenced their choice of patient order. In the control group, communication was the primary criterion, where participants tended to prioritize patients who could communicate and walk, categorizing them as "green" or less critical cases. In contrast, the experimental group, working in the VR environment, primarily focused on the severity of the injuries as the key factor, followed by the proximity of the patients to the responder.

**Fig. 2:**
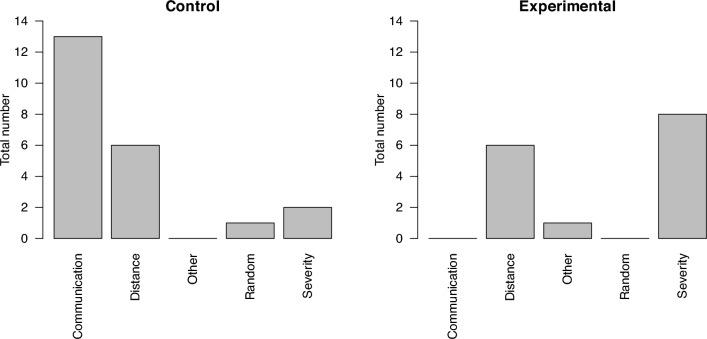
Patient prioritization by groups

The responses indicated that the participants in the experimental group often used a more systematic approach, typically starting from the most critical patient or the one closest to them and then moving in a circular pattern. This was partly due to limitations in the virtual environment, such as the inability to engage in natural communication with patients, which is a fundamental part of real-life triage. As a result, participants in the VR environment were required to rely on other visual cues and systematic strategies to assess the patients. The probands' preferences for how they prioritized individual patients for the control and experimental groups are shown in Fig. 2.

In conclusion, the hypothesis that the order of patient treatment would differ in the virtual environment compared to real-life scenarios was supported. The experimental group used severity and proximity as key factors, whereas the control group relied heavily on communication. This difference highlights the impact of the training environment on decision-making processes during triage.

## Discussion

This study aimed to assess the effectiveness of VR training for paramedic students in managing MCI compared to traditional training methods. The results highlighted several key findings that offer valuable insights into how immersive VR environments can influence and enhance the training process under high-pressure, real-world emergency conditions. By simulating complex scenarios, VR not only offers a controlled environment for skill development but also provides a unique platform for improving decision-making and response efficiency during critical incidents.

One of the primary advantages of VR training is its ability to create immersive, realistic environments that are difficult to replicate in conventional training settings [18]. Traditional training methods, which often involve classroom-based learning or mannequin-based simulations, provide foundational knowledge and skills but may lack the dynamic and emotional intensity of real-world scenarios. VR addresses this gap by enabling trainees to interact with lifelike situations, which can trigger more authentic emotional and cognitive responses [17]. In this study, VR’s immersive environment helped reduce cognitive load [19], as reflected by significantly lower mental demand and frustration levels in the experimental group. This suggests that VR can provide a more engaging and less stressful platform for high-intensity training, making it particularly useful in emergency preparedness.

The potential of VR extends beyond regular training cycles. In situations of crisis there is often a need for rapid and effective training for emergency responders [2] who may be deployed without sufficient preparation. VR provides an ideal solution in such circumstances. Its ability to simulate a wide range of emergency scenarios can enable quick yet comprehensive training, even for personnel with minimal prior experience. The structured, repeatable nature of VR training allows users to practice critical skills under pressure, making it a valuable tool for preparing responders in wartime or other crisis conditions, where traditional training resources may be scarce or insufficient. In these scenarios, VR offers a scalable and efficient solution to ensure that responders can be adequately prepared to handle real-time emergencies with minimal delay.

However, the study also highlighted certain limitations of VR training. Participants in the experimental group took significantly longer to complete tasks such as the METHANE report, likely due to the unfamiliarity of the VR interface and the need to adapt to virtual tools. In contrast, participants in the control group, who used traditional hands-on methods, were able to complete the tasks more quickly, possibly because they were more accustomed to interacting with physical objects. Despite the slower execution, VR participants showed higher accuracy in their reports, which suggests that the immersive environment may encourage more thoughtful decision-making and precision [20]. This finding has critical implications in emergency situations where accurate information can be as important as rapid response.

When comparing VR to conventional training, it is important to note that VR’s strength lies in its interactivity and ability to simulate a wide range of scenarios that evolve in real time. Traditional methods, while effective for basic skills training, often fail to replicate the complexity of real-world emergencies. VR, on the other hand, can present learners with dynamic, high-pressure situations that require rapid decision-making and adaptability—skills critical for emergency responders [6]. This study adds to the growing body of evidence that suggests VR can enhance not only technical skills but also emotional readiness for real-life challenges, making it a valuable tool in the training of medical professionals [21].

Unlike studies focusing on live or video-based simulations, such as Nieto Fernández-Pacheco et al. [15] and Curtis et al. [22], this research utilized a fully immersive VR environment to examine its impact on training outcomes for mass casualty incidents. Traditional live simulation approaches often rely on physical interaction with mannequins or actors to recreate realistic emergency scenarios. While these methods provide tactile feedback and dynamic interaction, they are inherently less consistent, as variables such as instructor-led cues or participant roles can vary across sessions. Additionally, compared to studies such as Kaim et al. [2] or Mirza et al. [23], which integrated augmented reality or haptic feedback, the VR system used in this research lacked these features, potentially impacting task execution speed and tactile interaction. Despite these limitations, the VR environment proved effective in enhancing accuracy in situational reporting, suggesting its potential for developing cognitive and decision-making skills under controlled conditions. This aligns with findings from Bednar et al. [17], who emphasized the role of VR in improving focus and precision during crisis simulations.

One of the limitations of this study lies in the exclusive use of the Valve Index VR system, which, despite its high level of immersion, posed challenges due to its reliance on handheld controllers. This system's controls introduced difficulties for some participants, particularly in managing virtual objects or transitioning between tasks efficiently, which may have affected their performance times. As VR technology continues to evolve, the integration of hand-tracking systems offers a clear path for improvement. Hand-tracking would significantly enhance user comfort by closely mimicking real-life interactions, allowing participants to focus more on the training content itself rather than struggling with the interface [24]. This shift would not only reduce the cognitive load associated with VR controllers but also streamline the learning process, making the technology more intuitive and effective for education and training. Additionally, augmented reality (AR) holds substantial potential for further enhancing immersive training experiences. By blending real-world and virtual elements, AR can provide users with a higher level of engagement, allowing them to interact with both digital and physical environments [2]. In comparison to findings by Kaim et al. [2], which demonstrated augmented and virtual reality's effectiveness in wartime training for rapid upskilling, this study similarly supports the utility of VR for fast response readiness, particularly in high-stakes MCI scenarios. While Kaim et al. emphasized the adaptability of VR for preparing reserve personnel with prior experience, these findings extend this advantage to paramedic students, showing VR's effectiveness in enhancing both cognitive and emotional readiness. However, unlike Kaim et al., whose study focuses on military conditions and combines both AR and VR to recreate the unique immediacy and stress of wartime environments, this research is set within a civilian, peacetime context, centering solely on VR's role in MCI training. This study specifically highlights VR's cognitive benefits, including reduced mental demand and frustration, and its positive impact on situational reporting accuracy, which contrasts with the AR-enhanced simulation of combat conditions used by Kaim et al. This distinction underlines VR's broader applicability for traditional emergency preparedness, while showing how AR may add additional layers of immersion beneficial for military training contexts. Further expanding VR and AR experiences with haptic feedback systems could elevate immersion even more by incorporating tactile sensations, which would help users gain a more comprehensive understanding of their training scenarios. Such advancements could deepen the educational impact by connecting cognitive learning with physical responses, ultimately enhancing overall training outcomes. Additionally, this research builds on earlier work [6] focused on scenario development within VR for paramedic training, advancing it by incorporating both an experimental and a control group to provide a robust comparative framework. Moreover, findings from recent studies suggest that combining AR and VR can increase immersion levels, potentially influencing key variables differently from a VR-only approach, though this remains speculative without further empirical data.

The sample size of 37 participants represents another limitation. This number reflects the majority of third-year paramedic students available for the study, although the total cohort was slightly larger. The limited sample size, dictated by the cohort's size, may restrict the generalizability of the findings and reduce the statistical power of some comparisons. Future studies should consider including participants from multiple institutions or additional cohorts to address this issue. Additionally, the voluntary nature of participation may have introduced selection bias, as students who chose to participate may have been more motivated or confident in their abilities. Furthermore, individual differences in prior VR experience may have influenced the results, with participants familiar with virtual environments potentially adapting more quickly to the interface.

Another limitation concerns also the generalizability of the findings beyond the specific context of this study. The results are based on a simulated mass casualty car accident scenario, which, while representative of many critical aspects of emergency response, may not fully capture the variability and unique challenges of other emergency scenarios, such as natural disasters or large-scale public health crises. Different VR systems or simulation designs could yield varied outcomes and may require further exploration.

Despite these limitations, the findings provide valuable insights into the potential benefits and drawbacks of using VR in medical and emergency training. Future research should aim to expand the sample size, explore different types of VR systems, and perhaps integrate more advanced haptic feedback or hand-tracking technologies to enhance the immersive experience and improve the precision of simulated training. Long-term studies are essential in evaluating how repeated exposure to VR environments influences skill retention and emotional resilience, particularly for emergency responders who operate in high-stakes, crisis-driven contexts.

VR’s ability to offer immersive, realistic simulations positions it as a powerful tool for rapidly training responders in scenarios where quick preparation is vital, such as during wartime or disaster events. The scalability and flexibility of VR make it an invaluable asset in these settings, ensuring that responders can be trained effectively and efficiently when time is of the essence. However, the integration of VR into training programs should be approached with the understanding that it is not intended to replace conventional training methods but to complement them. While VR systems are capable of autonomously tracking performance and providing objective evaluations, the role of expert-led debriefing remains crucial. Personalized feedback from experienced professionals allows trainees to reflect deeply on their performance, enabling them to refine their skills and decision-making capabilities in ways that VR evaluations alone may not capture. Thus, VR should be seen as a complementary component within the broader educational and training framework. When used in conjunction with traditional, hands-on training, VR can enhance learning outcomes by offering immersive, repeatable simulations. Each method—whether VR or conventional—contributes uniquely to skill-building and competency development, ensuring a more comprehensive approach to professional training and preparedness. This balanced combination ensures that learners are equipped not only with technical proficiency but also with the critical thinking and adaptability needed in real-world crises. Based on the findings of this study, the integration of VR into MCI training should focus on leveraging its unique strengths to complement existing methods. VR offers the ability to simulate environments and situations that are logistically or ethically challenging to recreate in traditional training, such as dynamically evolving incidents or scenarios with high psychological pressure. By incorporating VR as a preparatory tool, trainees can engage in iterative learning cycles, allowing them to refine their responses and decision-making processes before transitioning to live drills. This sequential approach ensures that VR enriches the overall training by targeting areas that are difficult to address through conventional techniques, while traditional methods provide the tactile and situational awareness needed for real-world application.

Current advancements in VR training for emergency response teams [25] indicate a promising direction for healthcare policy improvements [26]. VR, increasingly supported by AR and haptic feedback systems, allows responders to experience more comprehensive training environments that integrate tactile sensations with visual and auditory stimuli. This immersive approach has been shown to deepen the cognitive and physical learning impacts, enabling emergency personnel to better retain and apply skills in high-stress scenarios. In light of these benefits, it is recommended that healthcare policies begin to incorporate immersive training tools as part of standard emergency preparedness curricula [27]. This would enhance both the efficiency and accuracy of training outcomes, leading to better-prepared responders. For maximum policy impact, funding mechanisms and training standards should support the adoption of VR and AR [28], emphasizing scalability to ensure access across a range of institutions and responder levels. To further enhance the practical application of immersive training, pilot programs could focus on key areas such as triage in MCIs, situational reporting (e.g., METHANE protocol), decision-making under pressure, and handling aggressive patients [29]. These areas benefit greatly from the immersive and controlled environment that VR offers, enabling responders to practice critical skills in scenarios that closely mimic real-world challenges.

The successful implementation of VR training requires strategic planning and resource allocation. This includes investments in VR headsets, software platforms, and adapting training modules to align with local protocols. Funding opportunities, such as government grants or partnerships with technology providers, can significantly reduce initial costs. Starting with smaller, scalable pilot groups ensures the feasibility and long-term success of the program while providing evidence of its effectiveness.

## Conclusion

This study explored the potential of VR in training paramedic students for MCIs. The results suggest that VR can provide an immersive training environment, with indications of reduced mental demand and frustration among participants. Additionally, the study observed improved accuracy in situational reporting, which may reflect the potential of VR to support more deliberate and precise decision-making in controlled, high-pressure scenarios. However, the slower task completion times in the VR group point to the need for further refinement of VR interfaces to enhance usability and efficiency in similar settings.

Future advancements in VR technology, such as hand-tracking and haptic feedback, could improve user experience and the realism of training simulations. By integrating VR as a complementary method alongside traditional training approaches, emergency preparedness programs may better equip responders to navigate the complexities of real-world crises.

## Data Availability

The analyzed data will be made available to requesting researchers upon a reasonable request.
